# Artificial Intelligence-Driven Integrated Risk Assessment of Cardiovascular Disease (AIRA-CVD): A Technical Framework Incorporating Inflammatory Biomarker Signatures and Histopathological Vascular Remodeling

**DOI:** 10.7759/cureus.106615

**Published:** 2026-04-07

**Authors:** CFC Ogbuefi

**Affiliations:** 1 Department of Family Medicine, Federal University Teaching Hospital, Owerri, NGA

**Keywords:** artificial intelligence, cardiovascular disease, diagnostic informatics, digital health, health equity, inflammatory biomarkers, machine learning, predictive modeling, risk assessment, vascular remodeling

## Abstract

Cardiovascular disease (CVD) remains the leading cause of mortality in the United States, with persistent disparities driven by limitations in traditional risk stratification models and fragmented healthcare data systems. This conceptual technical report presents the Artificial Intelligence-Driven Integrated Risk Assessment of Cardiovascular Disease (AIRA-CVD) framework, a multimodal predictive architecture designed to integrate clinical diagnostic informatics, inflammatory biomarker signatures, and histopathological validation of vascular remodeling. The proposed framework leverages structured and unstructured electronic health record (EHR) data, cardiovascular imaging, and circulating biomarkers, including high-sensitivity C-reactive protein (hs-CRP) and interleukin-6 (IL-6), to generate individualized risk predictions. A distinguishing feature of AIRA-CVD is the incorporation of histopathological vascular remodeling as a ground-truth calibration standard, which is designed to enhance interpretability and is hypothesized to mitigate bias associated with traditional proxy-based models. The framework further integrates human-in-the-loop clinical oversight and continuous performance auditing to align with emerging ethical governance standards. By linking computational modeling with biological validation, AIRA-CVD is designed to provide a scalable and equity-centered approach to precision cardiovascular risk assessment and, if prospectively validated, may have the potential to contribute to improved clinical outcomes and reduced health disparities.

## Introduction

Cardiovascular disease (CVD) remains a leading contributor to morbidity, mortality, and healthcare expenditure in the United States and globally [1,2]. Despite advances in preventive strategies and therapeutic interventions, substantial disparities persist across racial, socioeconomic, and geographic populations, reflecting limitations in existing risk stratification frameworks.

Traditional models, including the Framingham Risk Score and pooled cohort equations, rely on a limited set of clinical variables and static population-level assumptions, often resulting in suboptimal predictive performance in diverse populations [3]. Recently, artificial intelligence (AI) and machine learning (ML) approaches have emerged as promising tools for improving cardiovascular risk prediction through the integration of high-dimensional datasets, including imaging, longitudinal clinical data, and electronic health records (EHRs) [4-6].

However, the rapid integration of AI into cardiovascular care has exposed critical challenges related to algorithmic bias, lack of interpretability, and insufficient regulatory oversight [7,8]. These limitations were previously discussed in our 2026 policy-oriented work [9], which provides a governance perspective but does not constitute empirical validation of the present framework. Existing AI models often rely on proxy variables such as healthcare utilization or cost, which may reflect systemic inequities rather than true disease burden, thereby reinforcing disparities.

While governance frameworks are essential, there remains a critical need for technically robust systems that operationalize these principles within clinical practice. To address this gap, we propose the Artificial Intelligence-Driven Integrated Risk Assessment of Cardiovascular Disease (AIRA-CVD) framework. This model integrates multimodal clinical data with inflammatory biomarker profiling and histopathological validation of vascular remodeling, thereby aligning predictive modeling with biological disease processes. The proposed framework is conceptual in nature and is intended to generate testable hypotheses regarding the integration of multimodal data, biological validation, and algorithmic fairness in cardiovascular risk prediction.

## Technical report

Study design and conceptual framework overview

This study presents a technical framework and conceptual model development for a multimodal AI-based cardiovascular risk prediction system. The AIRA-CVD architecture is designed as a layered system integrating clinical, molecular, and pathological data streams into a unified predictive engine.

Data sources and integration

Clinical Data (EHR-Based Informatics)

Structured and unstructured data are extracted from EHR systems, including demographics, vital signs, laboratory values, and cardiovascular imaging (echocardiography, computed tomography (CT) angiography). Natural language processing (NLP) models are applied to clinical notes to extract additional variables, improving data completeness and contextual understanding. Data standardization is achieved using established ontologies such as Systematized Nomenclature of Medicine Clinical Terms (SNOMED CT) and Logical Observation Identifiers Names and Codes (LOINC), enabling interoperability across systems.

Inflammatory biomarker integration

Inflammatory biomarkers are incorporated as core predictive features due to their central role in atherogenesis and vascular injury [10,11]. Key biomarkers include high-sensitivity C-reactive protein (hs-CRP), interleukin-6 (IL-6), tumor necrosis factor-alpha (TNF-α), and interleukin-1β (IL-1β) [10,11]. These are modeled as continuous, time-dependent variables to capture the cumulative inflammatory burden over time. Specifically, the model analyzes the synergistic effect of IL-6-mediated signaling on vascular remodeling, providing a more granular risk profile than traditional static lipid measurements. 

Target population and intended clinical setting 

The AIRA-CVD framework is designed to be used with adult patients who are getting their cardiovascular risk assessed in either an outpatient or hospital setting. The primary intended outcomes include prediction of major adverse cardiovascular events over a defined time horizon (e.g., 10-year atherosclerotic cardiovascular disease risk) and identification of high-risk phenotypes with subclinical vascular remodeling that may be under-recognized by traditional risk scores.

Histopathological validation and ground-truth labeling

A defining component of the AIRA-CVD framework is the integration of histopathological vascular remodeling (e.g., intimal thickening and plaque formation) as ground-truth data. Training datasets incorporate histological specimens from surgical and biopsy sources, imaging-pathology correlation datasets, and validated surrogate imaging markers. This approach ensures that predictive outputs are aligned with true biological disease progression rather than indirect proxies, with the aim of reducing reliance on proxy variables; whether this approach reduces algorithmic bias remains to be determined through empirical validation.

Workflow architecture and data pipeline

**Figure 1:**
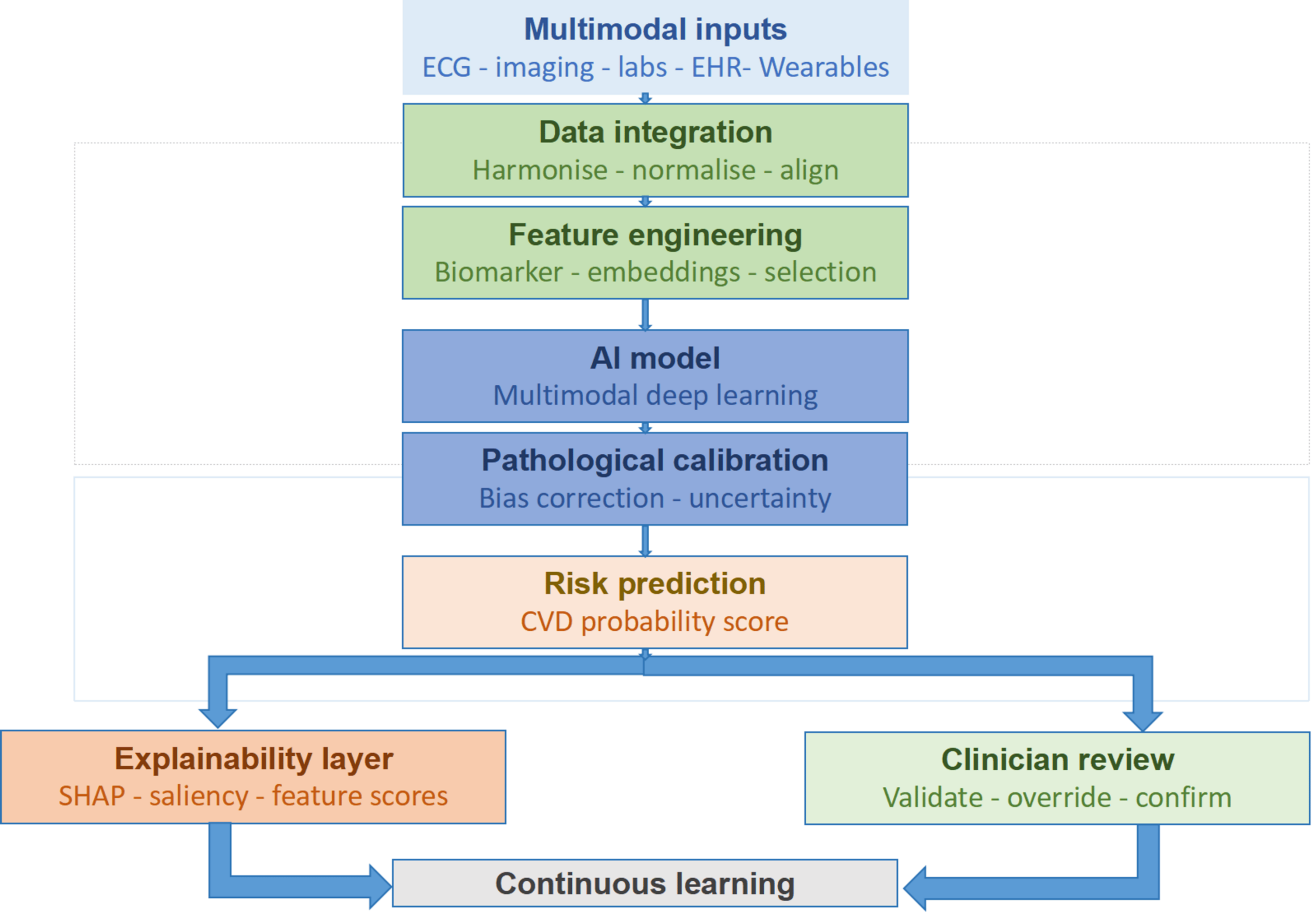
Conceptual architecture of AIRA-CVD Visual representation of the AIRA-CVD framework illustrating the integrated pipeline from multimodal data ingestion to clinician-validated risk prediction. The architecture highlights the feedback loop between the explainability layer and continuous learning modules. Image created manually by the author using WPS Office (Kingsoft, Beijing, China). AIRA-CVD: Artificial Intelligence-Driven Integrated Risk Assessment of Cardiovascular Disease; ECG: electrocardiogram; EHR: electronic health record; AI: artificial intelligence; CVD: cardiovascular disease; SHAP: SHapley Additive exPlanations

The AIRA-CVD pipeline follows a four-stage informatics workflow designed for clinical deployment as illustrated in Figure 1. In the ingestion stage, the system performs the extraction of multi-institutional EHR data via automated application programming interfaces (API). During preprocessing, laboratory values are standardized, and NLP-based feature extraction is applied to clinical documentation. In the analysis stage, a hybrid ensemble model processes the multimodal inputs, using convolutional neural networks (CNNs) to identify subclinical structural changes in imaging and transformer-based architectures for temporal data. Finally, the output stage generates an equity-adjusted risk score, accompanied by explainable AI visualizations for clinician review.

ML architecture and fairness

The AIRA-CVD system employs a hybrid ensemble modeling approach using gradient boosting machines for structured clinical data and CNNs for imaging analysis [4-6]. Transformer-based architectures are utilized for temporal and sequential data, such as longitudinal lab results and electrocardiogram (ECG) trends [6]. Model evaluation in future implementations is planned to include the area under the receiver operating characteristic curve (AUC) and Brier scores for calibration. To address algorithmic bias, the conceptual design incorporates stratified sampling, reweighting techniques for underrepresented populations, and fairness-aware optimization functions using metrics such as demographic parity difference and equal opportunity difference to promote equitable performance across demographic subgroups [7]. These AI cardiology approaches build on established deep learning techniques [12,13], leveraging big data methodologies commonly used in healthcare [14], including ECG analysis with AI [15].

Human-in-the-loop oversight

The framework integrates clinician oversight at critical decision points to ensure clinical safety and ethical accountability. Key features include XAI outputs using SHapley Additive exPlanations (SHAP) values to visualize feature contributions and clinician validation prior to high-risk classification [4,8]. Continuous feedback loops enable model recalibration based on real-world clinical outcomes and provider input.

Results

As this study presents a conceptual framework, the following represent anticipated system characteristics and theoretical advantages rather than empirically validated results. The AIRA-CVD model is designed to offer several potential system-level advantages over traditional risk prediction systems, as summarized in Table 1. These include the potential for improved predictive accuracy through multimodal data integration and biological validation, which may enhance discrimination and calibration beyond traditional static models [6]. They also encompass enhanced early detection of subclinical disease via inflammatory biomarkers and imaging. The framework is designed to mitigate algorithmic bias through histopathological ground truth and fairness optimization, particularly by addressing reliance on proxy variables associated with systemic inequities [7,8]. Finally, it is designed to provide improved clinical interpretability via explainable AI outputs and human oversight to build clinician trust.

**Table 1:** Comparison of established features in traditional models vs. proposed design goals of AIRA-CVD The AIRA-CVD column reflects proposed design characteristics and has not been empirically validated. AIRA-CVD: Artificial Intelligence-Driven Integrated Risk Assessment of Cardiovascular Disease; EHR: electronic health record; AI: artificial intelligence

Feature	Traditional models	AIRA-CVD framework
Data inputs	Limited clinical variables	Multimodal (EHR, imaging, biomarkers)
Temporal dynamics	Static	Dynamic
Biological validation	Absent	Histopathology-based
Bias mitigation	Minimal	Integrated
Interpretability	Limited	Explainable AI
Personalization	Population-based	Individualized

As an illustrative use case, AIRA-CVD is conceptually designed to enable the reclassification of a 55-year-old Black man with intermediate pooled cohort equation risk but elevated IL-6 levels and imaging evidence of early vascular remodeling into a higher risk category. In such a scenario, the framework would highlight inflammatory and structural features contributing to risk through explainable AI visualizations, thereby supporting the earlier initiation of preventive therapies and more intensive risk factor modification than would be suggested by traditional scores alone [12]. This scenario is hypothetical and intended solely to demonstrate the conceptual application of the framework and does not represent clinical evidence.

## Discussion

The AIRA-CVD framework represents a proposed advancement in cardiovascular risk prediction by integrating computational modeling with biological validation. Unlike traditional models, which rely primarily on static clinical variables, this conceptual approach incorporates dynamic, multimodal data streams that are expected to more accurately reflect disease progression, although this remains to be validated empirically.

A key innovation is the use of histopathological vascular remodeling as a ground-truth reference. This approach is intended to address limitations in current AI systems that rely on proxy variables, such as healthcare utilization, which may perpetuate systemic inequities. By anchoring predictions to biological evidence, the model is designed to enhance accuracy and fairness, although its efficacy remains to be validated empirically. 

The integration of inflammatory biomarkers further strengthens the model's ability to detect early disease processes, aligning with emerging evidence on the role of inflammation in cardiovascular pathology [10,11]. Additionally, the incorporation of human-in-the-loop oversight ensures that AI outputs remain clinically interpretable and ethically accountable, consistent with global governance recommendations [4,8].

From a public health perspective, this framework aligns with national priorities to reduce cardiovascular disease burden and health disparities. By enabling more precise and equitable risk stratification, AIRA-CVD has the potential to improve preventive care and optimize resource allocation [1,2].

In routine practice, AIRA-CVD could support clinicians in several ways if validated and deployed. For example, it may facilitate the earlier initiation of lipid-lowering and anti-inflammatory therapies in high-risk patients, guide more targeted use of imaging and invasive diagnostics based on individualized risk profiles, and provide equity-aware decision support that reduces the likelihood of underestimating risk in historically marginalized populations.

Limitations of this framework include the need for large, high-quality multimodal datasets and the technical challenges associated with integrating histopathological data at scale. Initial implementation is likely to be feasible only in tertiary centers and health systems with existing digital pathology, advanced imaging archives, and robust EHR infrastructure. In settings where histological specimens are not routinely available, the framework may need to rely on validated imaging-based surrogate markers of vascular remodeling, which could attenuate some of the anticipated benefits of biological ground-truthing. Future work should focus on prospective validation, development of multi-institutional consortia to support data sharing, phased real-world implementation, and alignment with evolving regulatory standards for AI-enabled clinical decision support tools.

## Conclusions

The AIRA-CVD framework provides a technically robust and clinically grounded conceptual approach to cardiovascular risk prediction by integrating AI with biological validation. Through the incorporation of inflammatory biomarkers and histopathological evidence, this proposed model seeks to address key limitations of existing risk assessment tools, including bias, lack of interpretability, and reliance on indirect measures of disease.

By aligning technical innovation with public health priorities and health equity principles, AIRA-CVD offers a scalable pathway that, if prospectively validated, may have the potential to contribute to improved cardiovascular risk stratification and inform strategies aimed at reducing health disparities. Future studies are required to evaluate the clinical validity, predictive performance, and real-world impact of the proposed framework.
